# The causal relationship between autoimmune diseases and age-related macular degeneration: A two-sample mendelian randomization study

**DOI:** 10.1371/journal.pone.0303170

**Published:** 2024-06-10

**Authors:** Linrui Li, Mingyue Zhang, Moxiu Gu, Jun Li, Zhiyuan Li, Rong Zhang, Chuanwang Du, Yun Lv

**Affiliations:** Department of Ophthalmology, Fushun People’s Hospital, Fushun, China. Sichuan Province, P.R. China; West Bengal University of Animal and Fishery Sciences, INDIA

## Abstract

**Objective:**

The aim of this study is to investigate the potential causal relationship between autoimmune diseases, including systemic lupus erythematosus, rheumatoid arthritis, inflammatory bowel disease, multiple sclerosis, and Type 1 diabetes, and age-related macular degeneration (AMD). By utilizing the two-sample Mendelian Randomization (MR) approach, we endeavor to address this complex medical issue.

**Methods:**

Genome-wide association study (GWAS) data for autoimmune diseases and AMD were obtained from the IEU Open GWAS database and the FinnGen consortium. A series of stringent SNP filtering steps was applied to ensure the reliability of the genetic instruments. MR analyses were conducted using the TwoSampleMR and MR-PRESSO packages in R. The inverse-variance weighted (IVW) method served as the primary analysis, complemented by multiple supplementary analyses and sensitivity tests.

**Results:**

Within the discovery sample, only a statistically significant inverse causal relationship between multiple sclerosis (MS) and AMD was observed (OR = 0.92, 95% CI: 0.88–0.97, *P* = 0.003). This finding was confirmed in the replication sample (OR = 0.85, 95% CI: 0.80–0.89, *P* = 3.32×10^−12^). No statistically significant associations were detected between systemic lupus erythematosus, rheumatoid arthritis, inflammatory bowel disease, and Type 1 diabetes and AMD.

**Conclusion:**

Strong evidence is provided by this study to support the existence of an inverse causal relationship between multiple sclerosis and age-related macular degeneration. However, no causal evidence was found linking other autoimmune diseases with AMD. These findings not only offer novel insights into the potential etiological mechanisms underlying AMD but also suggest possible directions for future clinical interventions.

## Introduction

Age-related macular degeneration (AMD) is an ophthalmic disease that increasingly poses a threat to global health. According to recent projections, by the year 2020, approximately 196 million individuals worldwide, constituting about 8.69% of the global population, were affected by AMD. Alarmingly, this figure is expected to surge to 288 million by the year 2040 [1]. The incidence of AMD is on an upward trajectory, particularly in economically disadvantaged and low human development index countries, thus contributing to exacerbating health disparities and imposing a disproportionate social and economic burden [2, 3].

AMD primarily targets the central visual area of the retina, which is crucial for everyday activities such as reading, driving, and facial recognition. The progression of the disease leads to a gradual deterioration of these functions, severely impacting the quality of life [4]. In addition to the considerable social and economic repercussions of AMD, recent research has also shifted its focus to explore its potential interactions and associations with other diseases, particularly autoimmune diseases such as systemic lupus erythematosus (SLE), rheumatoid arthritis (RA), inflammatory bowel disease (IBD), multiple sclerosis (MS), and Type 1 diabetes (T1D) [5–7]. These autoimmune diseases share the common characteristic of an overactive immune system attacking body tissues, while AMD is a complex disease associated with a myriad of factors including age, genetic predispositions, and environmental variables like smoking and unhealthy diet [8]. Contrary to traditional views positing that AMD is primarily driven by age, genetics, and environmental factors, recent evidence suggests that chronic inflammation and immune dysregulation may also be non-negligible components in the AMD disease course [9, 10].

This gap in our understanding prompts the investigation into whether a causal relationship exists between autoimmune diseases and AMD and, if so, the mechanisms underlying their interactions. However, most previous studies addressing this question have been observational in nature, which poses limitations due to their inability to eliminate confounding variables and reverse causality.

To address these limitations, the present study employs a two-sample Mendelian Randomization (MR) approach. By leveraging genetic variations as instrumental variables, the MR method aims to circumvent the issues of confounding and reverse causality inherent in traditional observational studies. These findings are anticipated to contribute to a more comprehensive understanding of the etiological mechanisms underpinning AMD and may offer new avenues for future clinical interventions targeting AMD and related autoimmune diseases.

## Materials and methods

### Study design

**Fig 1:**
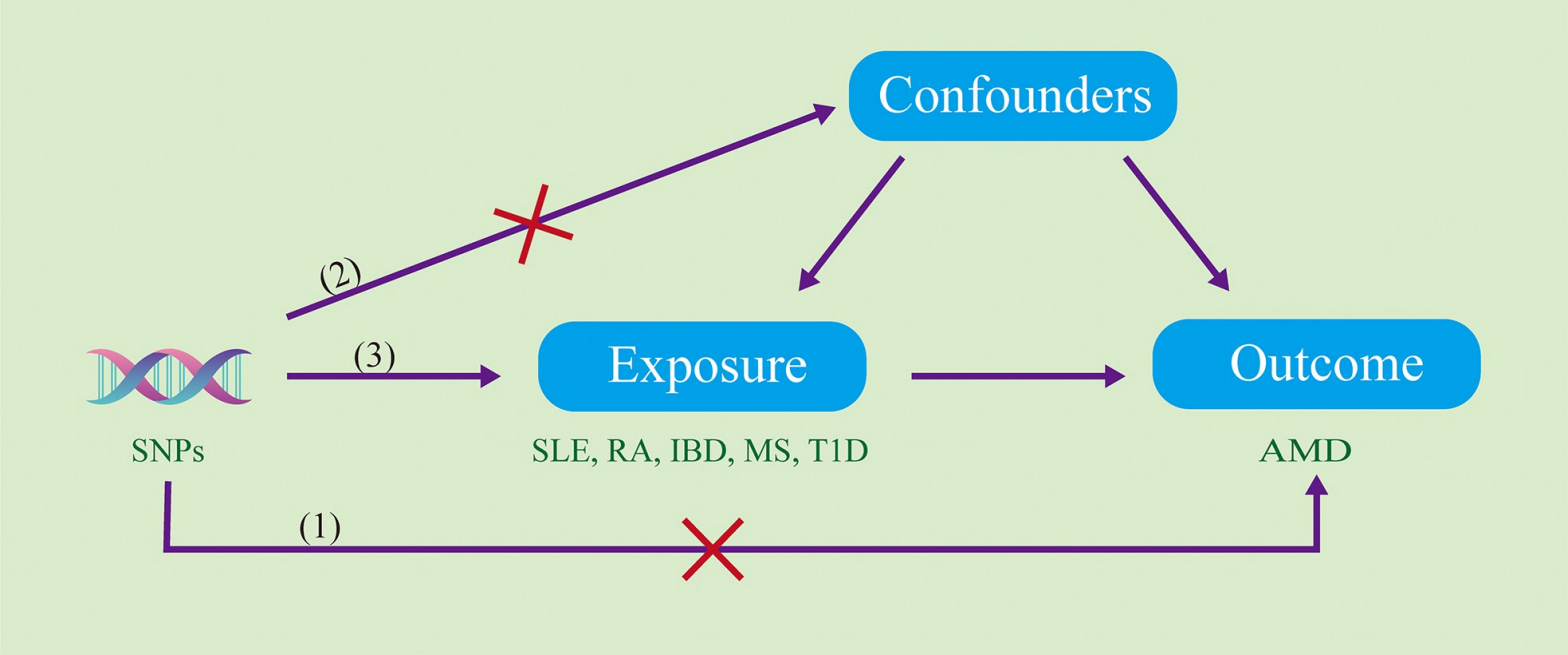
Schematic representation outlining the verification process for the three key assumptions underlying the two-sample MR analytical approach. The diagram illustrates steps for confirming the statistically significant associations of selected SNPs with autoimmune diseases, their independence from potential confounding factors, and their sole influence on AMD through their effect on autoimmune diseases.

The aim of the current study is to systematically investigate the causal relationship between autoimmune diseases and AMD utilizing a two-sample MR analytical approach. This method enables us to eliminate potential confounding factors, thereby allowing for a more accurate assessment of the association between the two. The execution of this MR study is predicated on three key assumptions: 1) The selected Single Nucleotide Polymorphisms (SNPs) must demonstrate statistically significant associations with autoimmune diseases. 2) The selected SNPs should be independent of any potential confounding factors. 3) The selected SNPs should influence age-related macular degeneration solely through their effect on autoimmune diseases. A detailed verification process for these assumptions is depicted in Fig 1. Data used in this study were sourced from multiple peer-reviewed, published studies, all of which obtained informed consent from participants and ethical approvals.

### Data source

The summary statistics for the genome-wide association studies (GWAS) on autoimmune diseases utilized in this study are primarily sourced from two authoritative databases: the IEU Open GWAS Project (https://gwas.mrcieu.ac.uk/) and the FinnGen Consortium (https://r9.finngen.fi/). Both databases predominantly feature samples of European descent. Specifically, data for the exposure group comprise 5,201 patients with SLE [11], 14,361 with RA [12], 12,882 with IBD [13], 14,498 with MS [14], and 6,683 with T1D [15]. Outcome variable data are derived from a pool of 3,763 patients suffering from AMD. To enhance the reliability and generalizability of our findings, replication samples were extracted from the FinnGen Consortium’s R9 dataset, including cases of SLE (1,023), RA (12,555), IBD (7,625), MS (2,182), and T1D (8,967). A comprehensive description of the data is presented in Table 1.

**Table 1:** Data sources and information for relevant exposures and outcomes.

Traits	Population	Sample size		Dataset ID
		Cases	Controls	
SLE^a^	European	5,201	9,066	ebi-a-GCST003156
RA^a^	European	14,361	43,923	ieu-a-832
IBD^a^	European	12,882	21,770	ieu-a-31
MS^a^	European	14,498	24,091	ieu-a-1025
T1D^a^	European	6,683	12,173	ebi-a-GCST005536
SLE^a^ (replication)	European	1,023	281,127	FinnGen ID: SLE_FG
RA (replication)	European	12,555	240,862	FinnGen ID: M13_RHEUMA
IBD (replication)	European	7,625	369,652	FinnGen ID: K11_IBD_STRICT
MS (replication)	European	2,182	373,987	FinnGen: G6_MS
T1D (replication)	European	8,967	308,373	FinnGen ID: finn-b-T1D_STRICT
AMD	European	3,763	205,359	FinnGen/GWAS ID:finn-b-H7_AMD

^a^ SLE: systemic lupus erythematosus; RA: rheumatoid arthritis; IBD: inflammatory bowel disease; MS: multiple sclerosis; T1D: type 1 diabetes.

### Selection of genetic instrumental variables

In this study, the reliability of the potential causal relationship between autoimmune diseases and AMD is ensured through a series of stringent SNP selection steps. Initially, only those SNPs meeting a genome-wide significance threshold of P < 5×10^−8^ were chosen as candidate genetic instruments, thereby assuring adequate statistical power. Secondly, to minimize the influence of linkage disequilibrium (LD), further filtering was conducted using a clustering method with an r^2^ value of less than 0.001 and a window width of 10,000 base pairs (kb). In the third step, a comprehensive query through the PhenoScanner database (http://www.phenoscanner.medschl.cam.ac.uk/phenoscanner) was employed to exclude SNPs associated with known confounding genes. Fourthly, the validity of the selected SNPs was further ascertained using the F-statistic as a criterion, calculated by the formula F = β^2^ / σ^2^ [16], where β represents the estimated effect of the SNP on the exposure variable and σ is the standard error of this effect. SNPs were confirmed to be devoid of weak instrument bias only if the F-statistic exceeded 10 [17]. Finally, to ensure consistency and accuracy of the results, palindromic SNPs and those missing in the outcome data set or exhibiting allele inconsistency between exposure and outcome (such as T/C and T/G) were excluded.

### Statistical analysis

In the MR analysis conducted in this study, all computations and model assessments were performed in the R software environment (Version 4.2.2), primarily utilizing the TwoSampleMR [18] and MR-PRESSO [19] packages. As the principal method for causal inference, the Inverse Variance Weighted (IVW) method was employed [20]. Additionally, MR-Egger, weighted median, weighted mode, and simple mode were also adopted as supplementary analysis methods to enhance the robustness of the results. To assess potential heterogeneity, Cochran’s Q test was implemented. When the p-value was less than 0.05, a random-effects model was utilized in the IVW-based MR analysis; otherwise, a fixed-effects model was employed [21]. Pleiotropy was assessed through MR-Egger regression, and the MR-PRESSO method was applied for the detection and exclusion of potential outliers. To further enhance the reliability of the results, a leave-one-out sensitivity analysis was conducted, aiding in the identification and exclusion of SNPs that might exert a significant impact on the overall results. Finally, during the multiple comparison adjustment, the Bonferroni method was employed, setting a p-value threshold of less than 0.005 as statistically significant.

## Results

### Instrumental Variables (IVs) for SNPs

Table 1 provides a detailed listing of the IVs utilized for various autoimmune diseases. By implementing a rigorous set of filtering criteria (*P* < 5 × 10^−8^, r^2^ < 0.001, window width kb = 10,000), the impact of LD was effectively minimized. Subsequent to this process, 45 SNPs were identified for SLE, 46 SNPs for RA, 62 SNPs for IBD, 49 SNPs for MS, and 36 SNPs for T1D. Further refinement was achieved through querying the PhenoScanner database, excluding palindromic SNPs, employing the MR-PRESSO method for outlier detection and removal, and conducting a leave-one-out sensitivity analysis. As a result, 34 SNPs were ultimately selected as IVs for SLE, 37 SNPs for RA, 62 SNPs for IBD, 49 SNPs for MS, and 36 SNPs for T1D for inclusion in the MR analysis.

### SNP selection in replication samples

Utilizing a similarly rigorous set of SNP filtering procedures, as applied to the discovery samples, further selection was conducted in the replication samples. Specifically, by adhering to analogous P-value thresholds, LD criteria, and other relevant metrics, varying numbers of SNPs were ultimately retained in the replication samples as IVs for different categories of autoimmune diseases. More precisely, 7 relevant SNPs were retained for SLE, 22 for RA, 40 for IBD, 5 for MS, and T1D. For a detailed list of SNPs and related information, please refer to Table 2.

**Table 2:** Excluded SNPs and the reasons for removing these SNPs in discovery samples.

Exposure	Exclude the effect of LD	PhenoScanner manually excluded	Removed palindromic SNPs	MRPRESSO method to identify and remove outliers	Leve-one-out analysis	nSNPs (Percentage of missing data)
SLE^a^	45	rs389884, rs12524498	rs28361029, rs2736332, rs28834423	NA	rs4274624, rs7823055, rs10048743, rs13332649, rs58721818, rs597808	34 (24.4%)
RA^a^	46	NA	rs13330176, rs225433, rs2661798, rs34536443, rs3799963, rs4452313	rs6936656, rs62395855, rs61828284	NA	37 (19.6%)
IBD^a^	62	NA	NA	NA	NA	62 (0)
MS^a^	49	NA	NA	NA	NA	49 (0)
T1D^a^	36	NA	NA	NA	NA	36 (0)

^a^ SLE: systemic lupus erythematosus; RA: rheumatoid arthritis; IBD: inflammatory bowel disease; MS: multiple sclerosis; T1D: type 1 diabetes; LD: linkage disequilibrium; SNP: single nucleotide polymorphism.

### Results of MR analysis on the influence of autoimmune diseases on AMD

**Fig 2:**
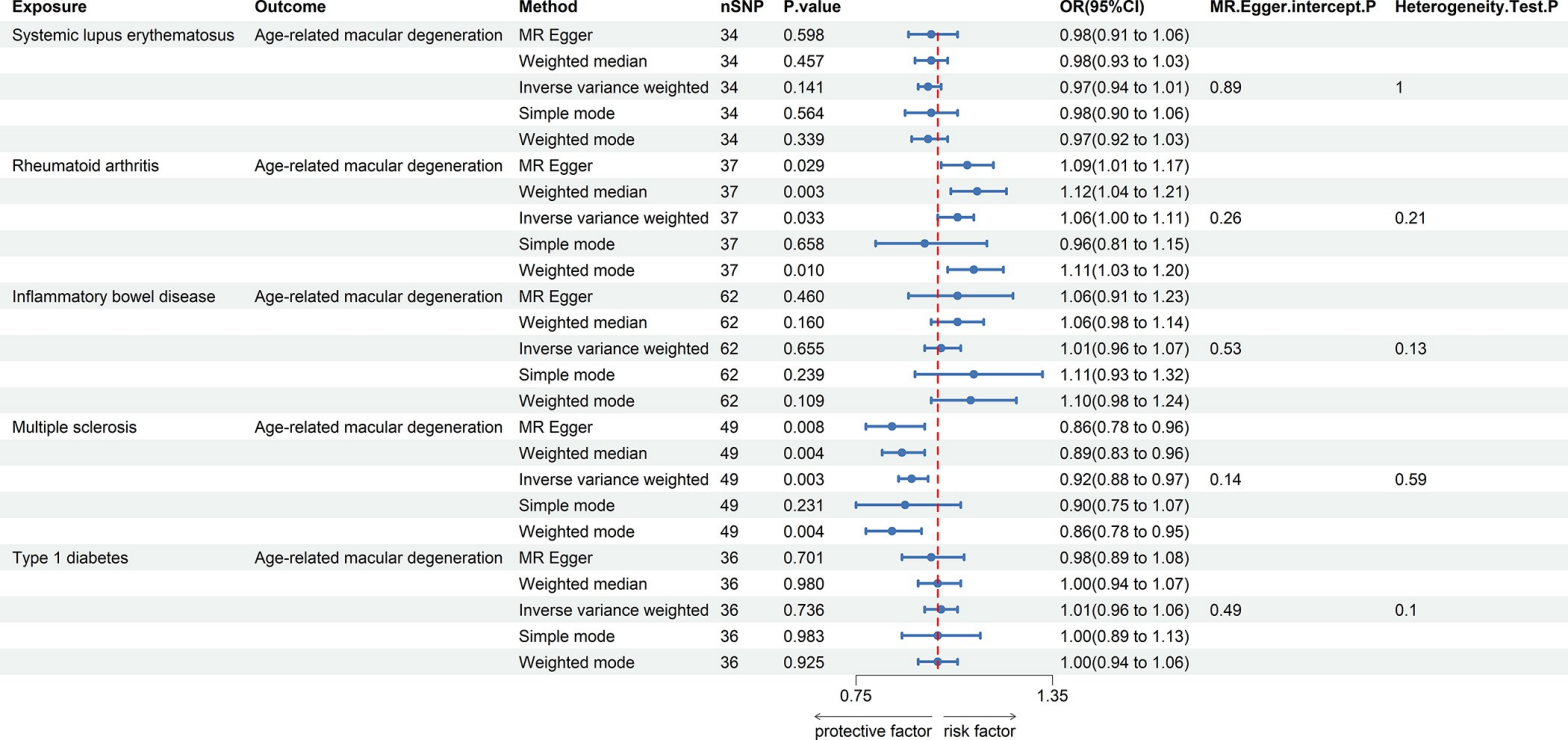
Forest plot illustrating MR analysis results for the association between autoimmune diseases and AMD in the discovery sample.

Fig 2 and the associated S1-S5 Figs in S1 File present the outcomes of the MR analysis between autoimmune diseases and AMD. After conducting a fixed-effects IVW analysis, a negative correlation between SLE and AMD was observed (OR = 0.97, 95% CI: 0.94–1.01, *P* = 0.141). However, this association lacked statistical significance, implying no causal relationship between the two conditions. Similarly, a positive correlation was noted between RA and AMD (OR = 1.06, 95% CI: 1.00–1.11, *P* = 0.033), but it failed to meet the threshold for statistical significance, thus precluding any conclusions about causality. For IBD and AMD, the fixed-effects IVW analysis also did not reveal a significant association (OR = 1.01, 95% CI: 0.96–1.07, *P* = 0.655). In contrast, a statistically significant negative correlation between MS and AMD (OR = 0.92, 95% CI: 0.88–0.97, *P* = 0.003) was maintained even after Bonferroni correction, suggesting a potential negative causal relationship between the two. No statistically significant correlation was found between T1D and AMD (OR = 1.01, 95% CI: 0.96–1.06, *P* = 0.736). The robustness of these MR analyses was supported through the application of MR-PRESSO to exclude outliers in causal estimates. Furthermore, MR-Egger intercept analysis did not indicate significant directional pleiotropy, and tests for heterogeneity did not reveal any underlying variability (Fig 2). These results offer preliminary yet critical insights into the potential causal relationships between autoimmune diseases and AMD, particularly concerning MS and AMD.

### Replication sample analysis

**Fig 3:**
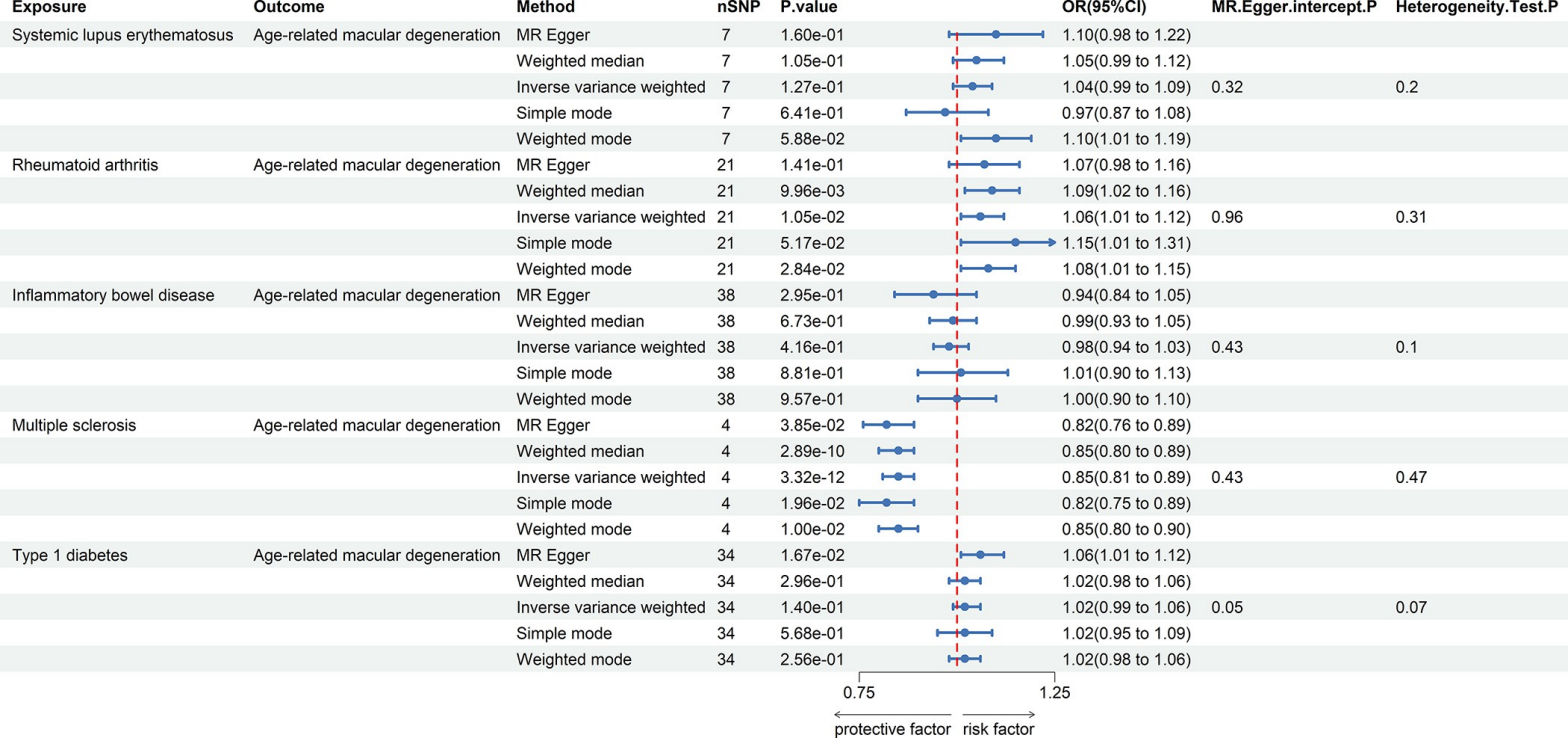
Forest plot illustrating MR analysis results for the association between autoimmune diseases and AMD in the replication sample.

In the analysis of the replication sample, the same methodologies employed in the discovery sample were adhered to (Table 3). Results from the replication sample further substantiated the previously observed negative causal relationship between MS and AMD. Specifically, a fixed-effects IVW analysis revealed a statistically significant association between MS and AMD (OR = 0.85, 95% CI: 0.80–0.89, *P* = 3.32×10^−12^), with the direction of effect being consistent with the discovery phase (see Fig 3 and S6-S10 Figs in S1 File). Concurrently, the findings in the replication sample reconfirmed the absence of statistically significant associations between SLE, RA, IBD, and T1D with AMD (*P*>0.05), thereby reinforcing the notion that no causal relationships exist between these conditions and AMD. The successful replication phase bolsters our confidence in the negative causal relationship between MS and AMD, providing more robust evidence. For other autoimmune diseases, such as SLE, RA, IBD, and T1D, the congruent results from two independent samples further strengthen our confidence in ruling out any causal relationships with AMD.

**Table 3:** Excluded SNPs and the reasons for removing these SNPs in replication samples.

Exposure	Exclude the effect of LD	PhenoScanner manually excluded	Removed palindromic SNPs	MRPRESSO method to identify and remove outliers	Leve-one-out analysis	nSNPs (Percentage of missing data)
SLE^a^	7	NA	NA	NA	NA	7 (0)
RA^a^	22	NA	NA	rs62405652	NA	21 (4.8%)
IBD^a^	40	NA	rs4795395	rs111432088	NA	38 (5%)
MS^a^	5	NA	rs1034321	NA	NA	4 (20%)
T1D^a^	36	NA	rs1131017, rs9276587	rs9276587	NA	34 (5.6%)

^a^ SLE: systemic lupus erythematosus; RA: rheumatoid arthritis; IBD: inflammatory bowel disease; MS: multiple sclerosis; T1D: type 1 diabetes; LD: linkage disequilibrium; SNP: single nucleotide polymorphism.

## Discussion

This study systematically analyzed the potential causal relationship between autoimmune diseases (SLE, RA, IBD, MS, and T1D) and AMD using two distinct MR methods. Genetic IVs were rigorously selected, and the associations were scrutinized through multiple adjustments and sensitivity analyses. Our findings suggest a potential negative causal relationship between MS and AMD, a relationship that was further corroborated in a replication sample, thereby enhancing the reliability of this observation. However, it should be noted that no clear causal relationships were identified between SLE, RA, IBD, T1D, and AMD.

These findings prompted an in-depth investigation into the possible mechanisms, particularly considering the role of interferon-beta (IFN-β) in the treatment of MS [22]. IFN-β was observed to influence multiple molecules and pathways related to immune cell migration and activation. In the context of MS, expression of adhesion molecules VLA-4 and VCAM is reduced by IFN-β, potentially decreasing immune cell aggregation at the blood-brain barrier [23]. This is particularly intriguing since AMD also involves immune cells in the retina and choroid, notably microglia and monocytes, which play key roles in angiogenesis [24]. Existing research has indicated that microglia and monocytes promote vascularization in animal models of AMD [25]. Therefore, IFN-β may suppress AMD progression by reducing the aggregation and activity of these cells in the retina. This hypothesis aligns with the findings of Luckoff et al., who demonstrated that IFN-β and its receptor IFNAR play crucial roles in the activation and infiltration of retinal microglia/macrophages [25]. Moreover, IFN-β’s ability to induce apoptosis in memory B cells through the Fas receptor/TACI pathway [22, 26] may also have therapeutic implications for AMD. Since AMD is generally an age-related disease often accompanied by chronic inflammation and immune activation in the elderly, IFN-β could exert anti-AMD effects by reducing chronic inflammation and immune cell activation [27, 28]. Nonetheless, the potential side effects of IFN-β, such as flu-like symptoms and muscle pain, cannot be overlooked, especially in older patients with AMD. Thus, further safety studies may be warranted before IFN-β can be employed in AMD treatment, potentially in combination or dosage adjustment with other therapies such as anti-VEGF treatments [29]. Therefore, careful consideration of the risks and benefits is essential when contemplating the use of IFN-β for AMD therapy, possibly necessitating dosage optimization and patient selection. Another avenue for future research could be the development of more selective IFN-β analogs or antagonists targeting specific receptors or signaling pathways to minimize side effects while maintaining or enhancing therapeutic efficacy. Overall, due to its pleiotropic effects and broad regulatory influence on the immune system, IFN-β holds therapeutic potential across a range of neuro-immunological diseases, including MS and AMD. However, to fully realize and apply this potential, further mechanistic research and clinical trials are needed, with particular attention to resolving issues related to adverse effects, to make it more suitable for diverse patient populations.

In addition to the previously discussed findings, the complement system plays a pivotal role in both AMD and MS, albeit with distinct functionalities in each disease. In AMD, complement activation is primarily localized to the formation of membrane attack complexes in the aging choroid [30]. In MS, however, the role of the complement system is more complex, encompassing inflammation in the central nervous system (CNS) and synaptic pruning [31]. It is essential to emphasize the dual nature of the complement system, participating in normal immune responses while also having the potential to induce excessive immune activity and tissue damage. In AMD, vascular loss in the choroid is considered a significant etiological feature [32]. Conversely, in MS, the complement system may also play a role due to its association with the blood-brain barrier and the imperative of vascular health in preventing the entry of complement proteins into the CNS [33]. Given the observed hyperactivity of the complement system in both AMD and MS, complement inhibitors could have potential therapeutic value in the treatment of these diseases. The critical question, however, lies in determining which part of the complement pathway should be inhibited and at which stage of disease progression. While AMD and MS are two distinct diseases involving different physiological systems, they share some commonalities in complement activation and etiology. These similarities may provide useful clues for the development of novel treatment approaches for both diseases. However, to substantiate these hypotheses, further empirical research is needed. Such studies may include in-depth molecular mechanistic research to understand the precise roles of complement in these diseases and clinical trials to evaluate the efficacy and safety of complement inhibitors as potential therapeutic strategies.

In addition to MS, it is crucial to acknowledge that the role of the complement system may vary across different autoimmune diseases when exploring the relationship with AMD. In RA, the complement system is likely primarily involved in joint inflammation and tissue destruction [34, 35], while in T1D, it may be associated with the autoimmune destruction of pancreatic β cells [36]. This could suggest that the pathways involving the complement system in these diseases might not significantly overlap with the pathological processes observed in AMD, where complement dysregulation primarily contributes to retinal degeneration. These distinctions could partially explain why these diseases do not exhibit significant genetic or pathological associations with AMD. Furthermore, each autoimmune disease presents unique immune and inflammatory response characteristics. For instance, SLE may involve a broad autoimmune response [37], which, while systemic, does not directly impact the retinal cells as seen in AMD. In contrast, IBD is associated with local intestinal inflammation and could have secondary effects on the eye such as episcleritis, uveitis, and scleritis, which are distinct from the pathogenesis of AMD [38]. These immunological disparities might contribute to the asynchronous pathogenesis of these diseases with AMD. Additionally, the onset of autoimmune diseases is likely influenced by both genetic variations and environmental factors, whose roles may differ across various diseases. For instance, environmental triggers such as UV exposure and smoking might play a more significant role in AMD compared to autoimmune diseases like T1D, where genetic factors and early life exposure to certain viruses are more impactful [39]. Moreover, the high heterogeneity of autoimmune diseases could be one reason for the difficulty in detecting their associations with AMD. Consequently, future research should focus on in-depth exploration of the immune regulatory mechanisms and inflammatory pathways of these diseases, and how they interact with the development mechanisms of AMD. Understanding these interactions at a molecular level could provide insights into the lack of significant associations and guide targeted therapeutic strategies for AMD in patients with these autoimmune diseases. More precise molecular level studies may uncover subtle links between these diseases and AMD. Although current research has not definitively established direct links between other autoimmune diseases and AMD, this does not preclude their potential complex interplay in pathophysiology. Additional basic and clinical research is needed to unravel the nature of these complex relationships, providing valuable insights for future therapeutic strategies.

This study has made preliminary strides in exploring the potential causal relationships between MS and AMD, yet it comes with several limitations. Firstly, despite rigorous selection criteria for SNPs as genetic markers, potential biases could still exist, or these markers might not comprehensively reflect the genetic diversity of the overall population. Secondly, although MR provides initial clues about causality, it does not delve into the underlying biological mechanisms. Lastly, previous research indicates that IFN-β holds some promise in AMD treatment [40], but its safety and long-term efficacy in clinical settings still require further investigation. To overcome these limitations, future research directions could include: 1) Further exploration of the genes and biological mechanisms to comprehensively understand potential causal relationships between MS and AMD. 2) A thorough assessment of the safety and side effects of IFN-β and other potential treatment options, such as complement inhibitors. 3) Conducting multi-center, multi-ethnic clinical trials to enhance the reliability and applicability of research findings. Through these comprehensive research measures, we hope to more precisely elucidate the complex relationship between MS and AMD and potentially develop more effective treatment modalities.

In conclusion, our study comprehensively evaluated the potential causal relationships between multiple autoimmune diseases and AMD through the use of two-sample MR methods. The results indicate a negative causal association between MS and AMD, a relationship consistently corroborated in replication cohorts. However, no statistically significant causal relationships were observed between other autoimmune diseases like SLE, RA, IBD, and T1D with AMD. These findings not only offer a novel perspective on the etiological basis of AMD but may also have a positive impact on future clinical interventions targeted at AMD. Additionally, this study explored the roles that IFN-β and the complement system may play in the interactions between MS and AMD, providing valuable clues for subsequent mechanistic research and targeted therapies.

## Supporting information

S1 File (DOCX)

S1 Data (XLSX)
