# Supplementary material for: The causal relationship between autoimmune diseases and age-related macular degeneration: A two-sample mendelian randomization study
Source: PLoS One. 2024 Jun 10;19(6):e0303170. doi: 10.1371/journal.pone.0303170 (PMC11164335; doi:10.1371/journal.pone.0303170)
Supplement: S1 File — (DOCX) [file pone.0303170.s001.docx]

Supplementary material

**Fig S1.** Causal relationship between SLE and AMD visualized through multiple analysis techniques including scatter plot, forest plot, leave-one-out plot, and funnel plot in the discovery sample.

**Fig S2.** Causal relationship between RA and AMD visualized through multiple analysis techniques including scatter plot, forest plot, leave-one-out plot, and funnel plot in the discovery sample.

**Fig S3.** Causal relationship between IBD and AMD visualized through multiple analysis techniques including scatter plot, forest plot, leave-one-out plot, and funnel plot in the discovery sample.

**Fig S4.** Causal relationship between MS and AMD visualized through multiple analysis techniques including scatter plot, forest plot, leave-one-out plot, and funnel plot in the discovery sample.

**Fig S5.** Causal relationship between T1D and AMD visualized through multiple analysis techniques including scatter plot, forest plot, leave-one-out plot, and funnel plot in the discovery sample.

**Fig S6.** Causal relationship between SLE and AMD visualized through multiple analysis techniques including scatter plot, forest plot, leave-one-out plot, and funnel plot in the replication sample.

**Fig S7.** Causal relationship between SLE and AMD visualized through multiple analysis techniques including scatter plot, forest plot, leave-one-out plot, and funnel plot in the replication sample.

**Fig S8.** Causal relationship between SLE and AMD visualized through multiple analysis techniques including scatter plot, forest plot, leave-one-out plot, and funnel plot in the replication sample.

**Fig S9.** Causal relationship between SLE and AMD visualized through multiple analysis techniques including scatter plot, forest plot, leave-one-out plot, and funnel plot in the replication sample.

**Fig S10.** Causal relationship between SLE and AMD visualized through multiple analysis techniques including scatter plot, forest plot, leave-one-out plot, and funnel plot in the replication sample.

**
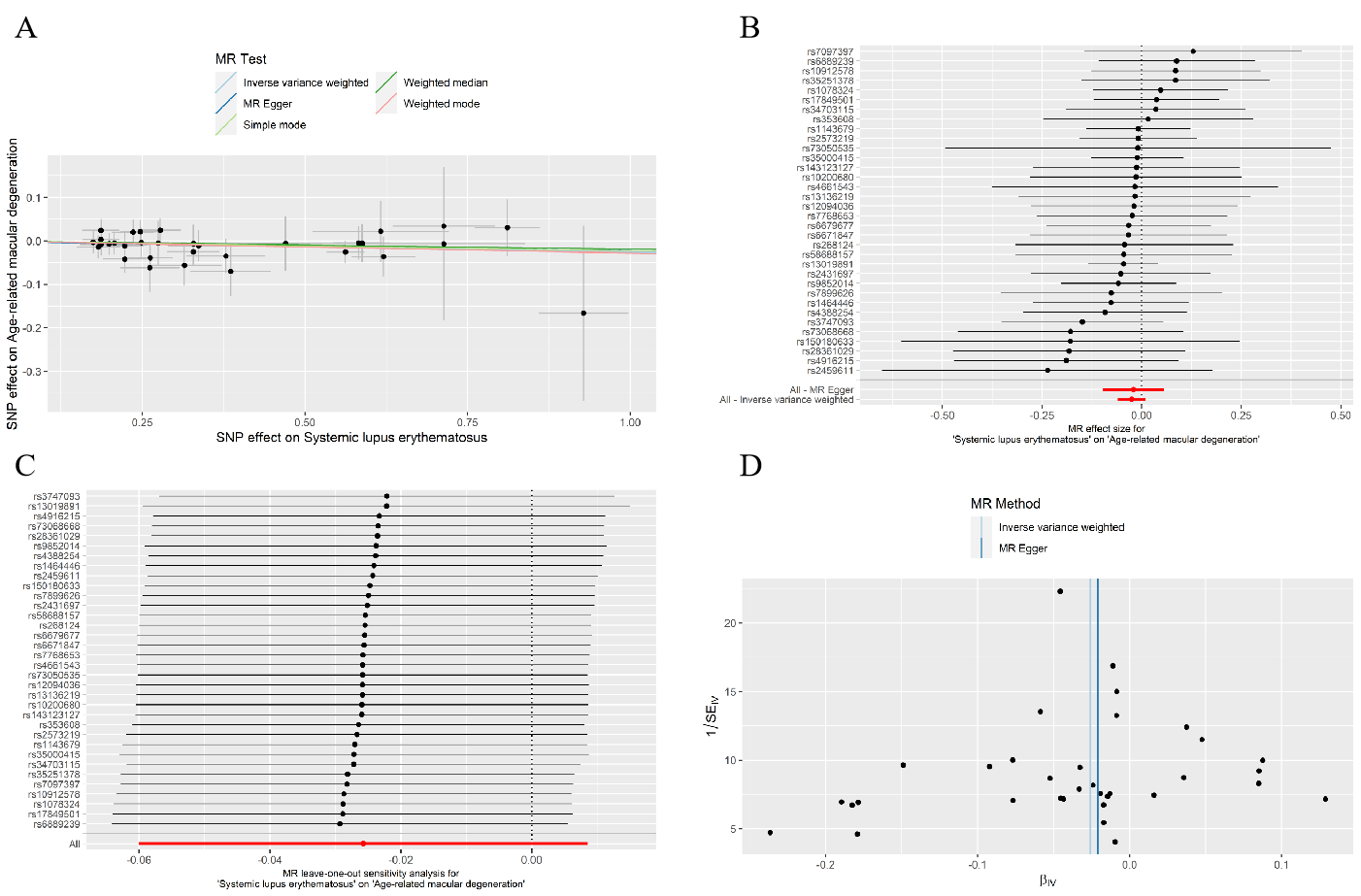
**

**Fig S1.** Causal relationship between SLE and AMD visualized through multiple analysis techniques including scatter plot, forest plot, leave-one-out plot, and funnel plot in the discovery sample.

**
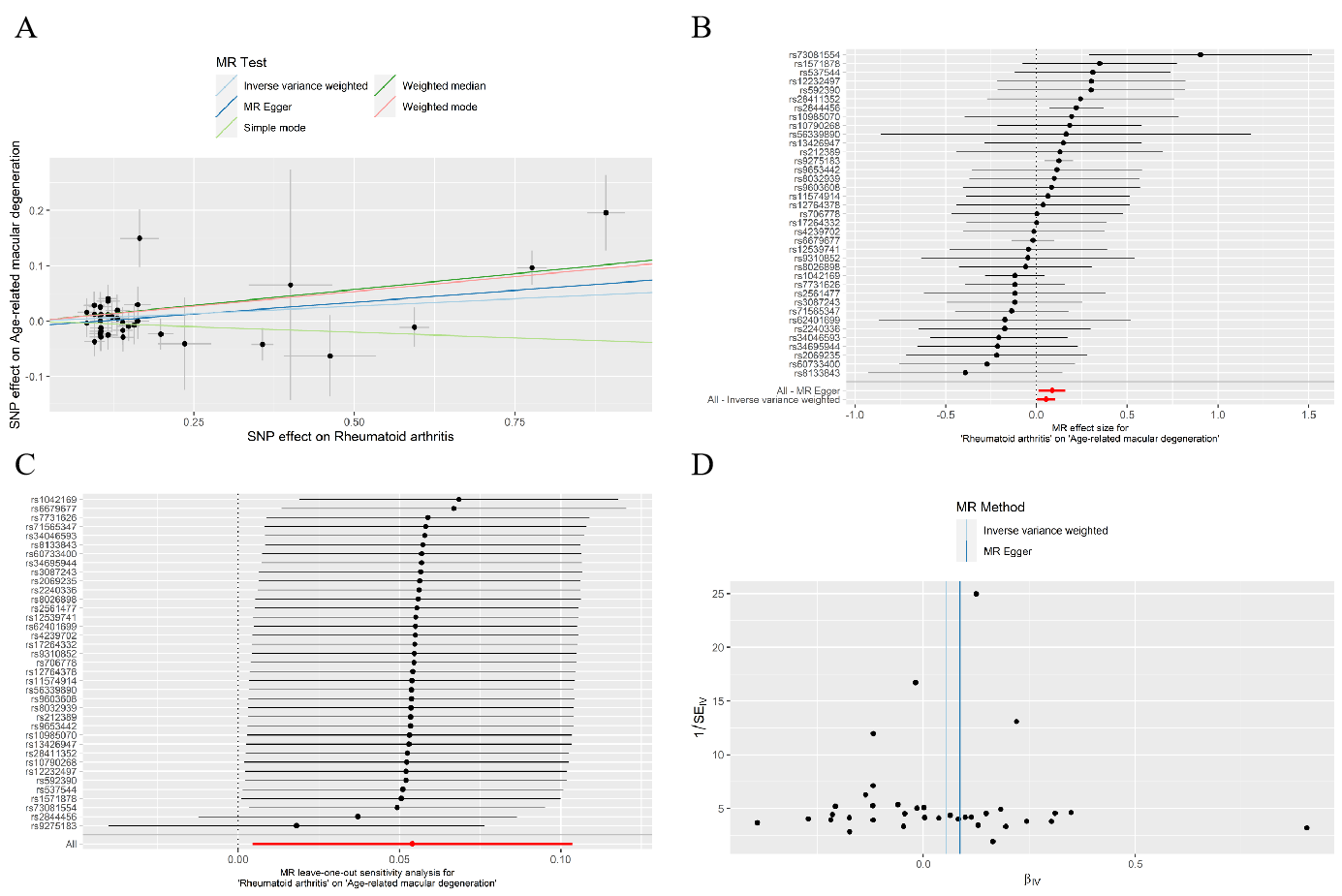
**

**Fig S2.** Causal relationship between RA and AMD visualized through multiple analysis techniques including scatter plot, forest plot, leave-one-out plot, and funnel plot in the discovery sample.

**
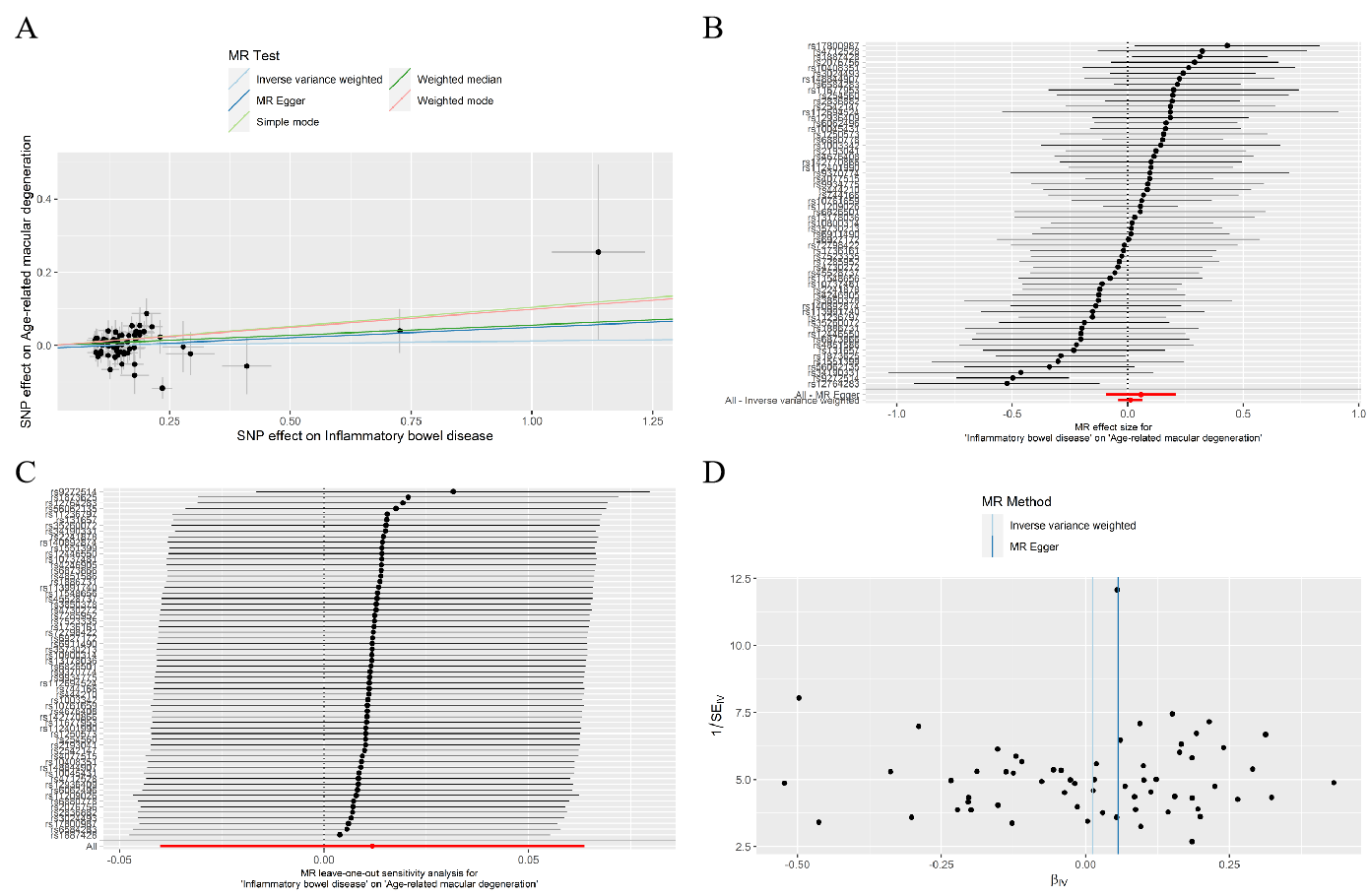
**

**Fig S3.** Causal relationship between IBD and AMD visualized through multiple analysis techniques including scatter plot, forest plot, leave-one-out plot, and funnel plot in the discovery sample.

**
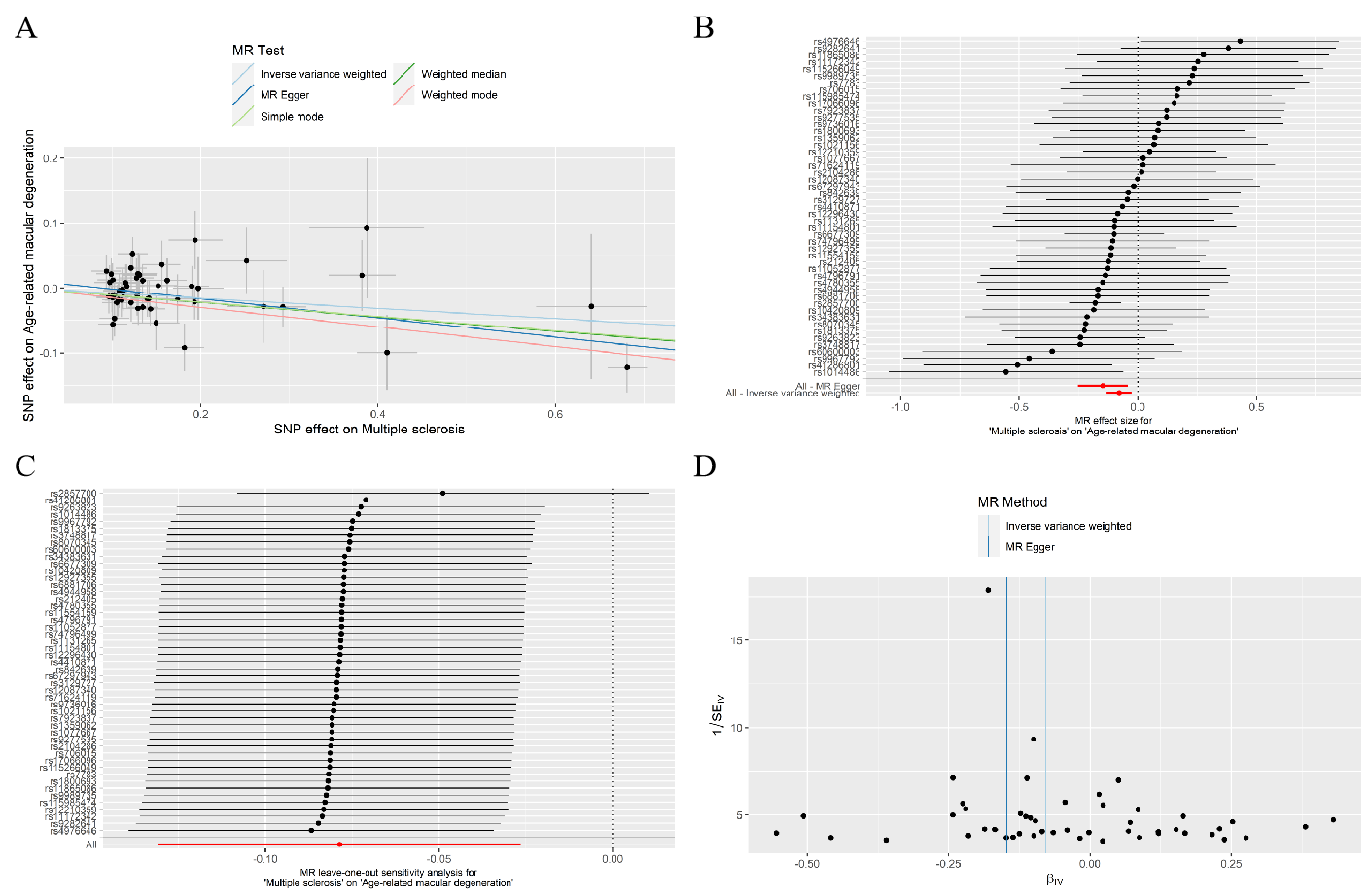
**

**Fig S4.** Causal relationship between MS and AMD visualized through multiple analysis techniques including scatter plot, forest plot, leave-one-out plot, and funnel plot in the discovery sample.

**
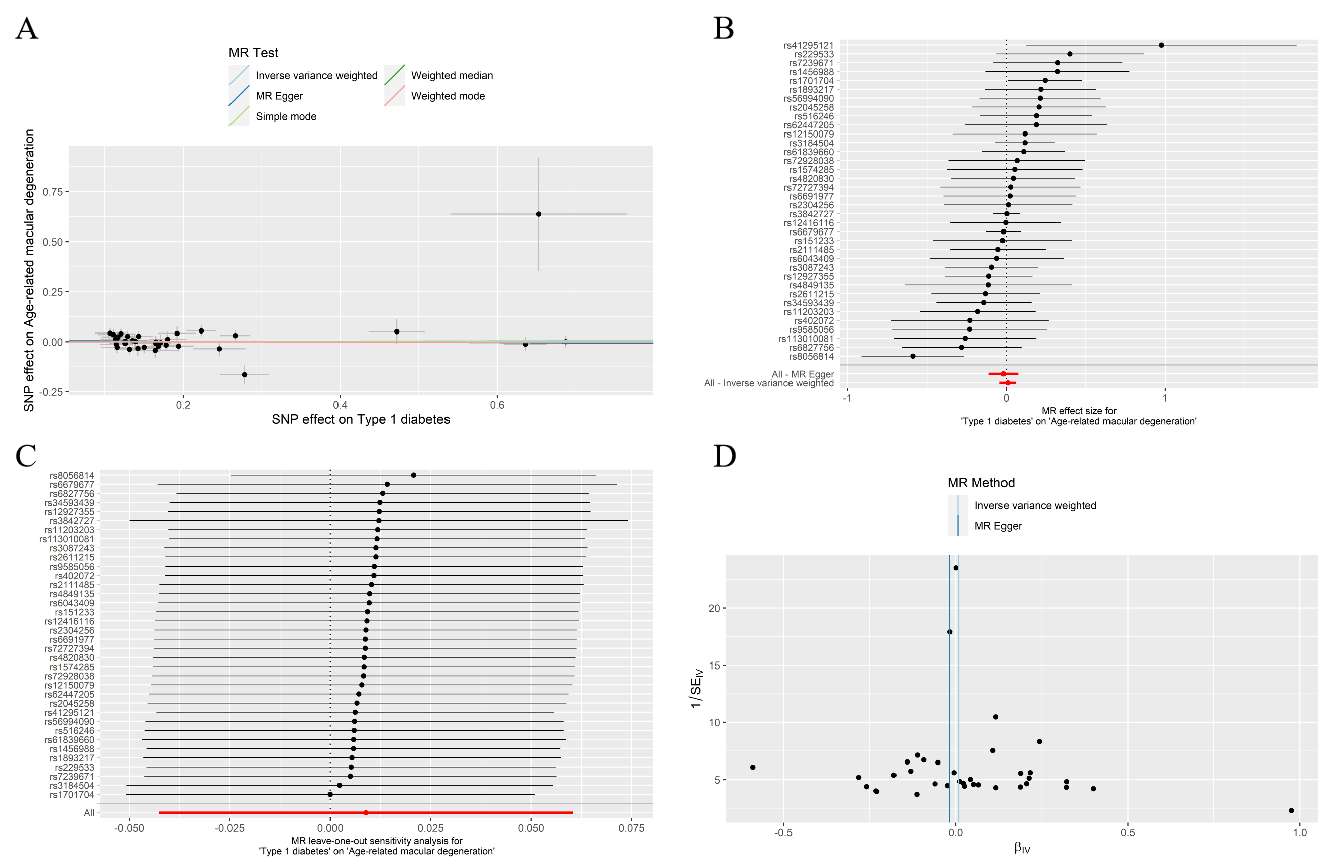
**

**Fig S5.** Causal relationship between T1D and AMD visualized through multiple analysis techniques including scatter plot, forest plot, leave-one-out plot, and funnel plot in the discovery sample.

**
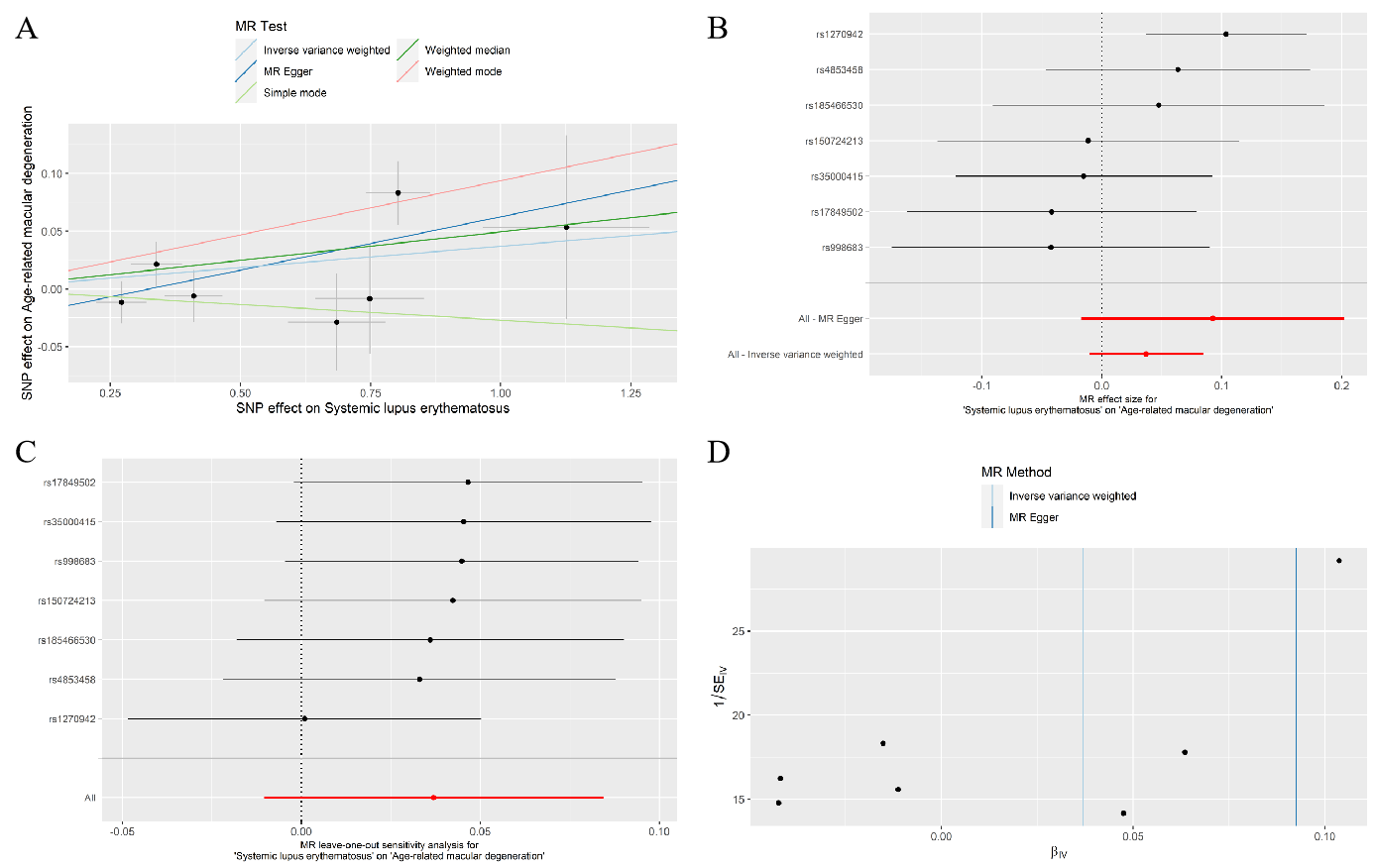
**

**Fig S6.** Causal relationship between SLE and AMD visualized through multiple analysis techniques including scatter plot, forest plot, leave-one-out plot, and funnel plot in the replication sample.

**
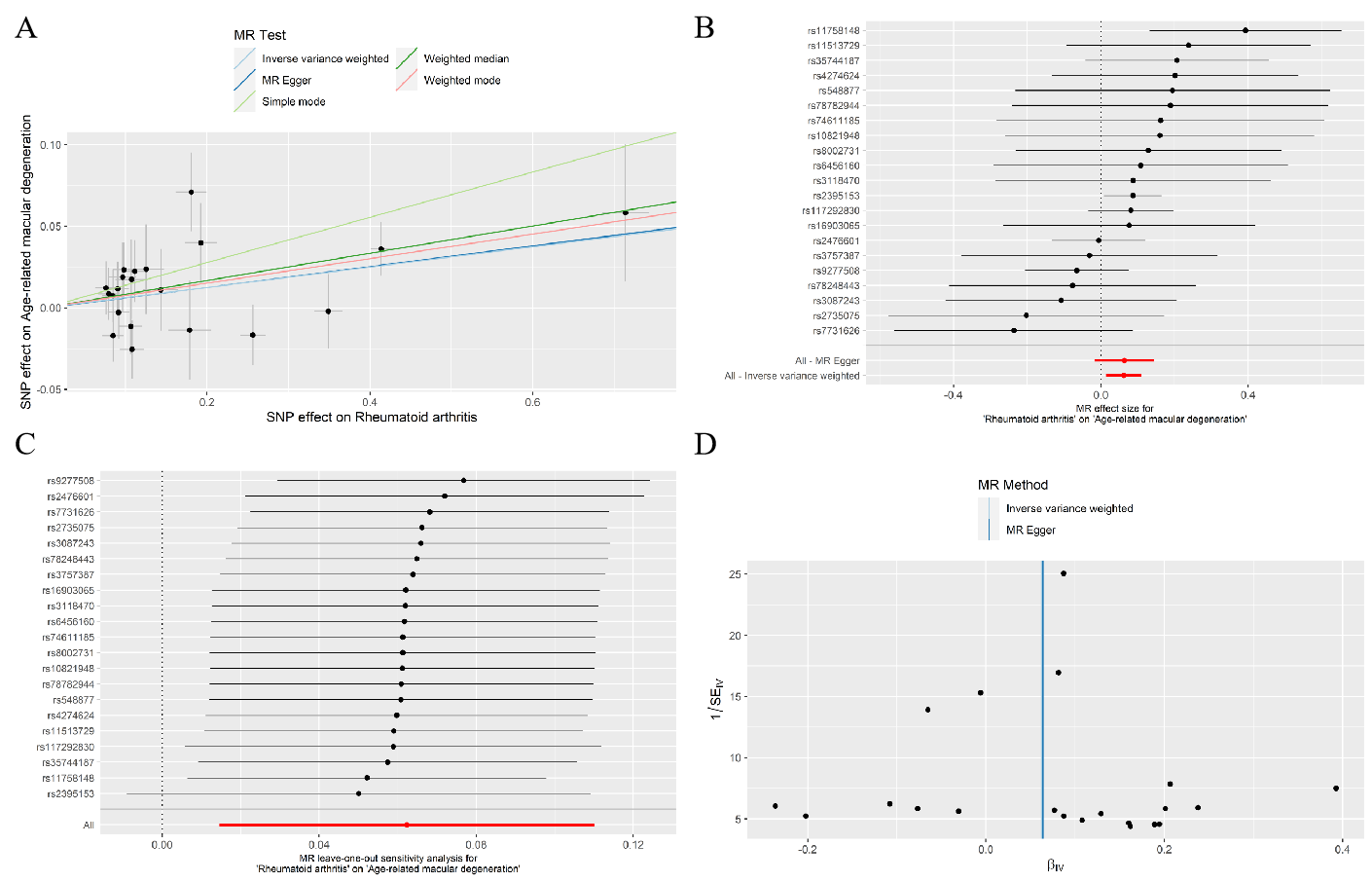
**

**Fig S7.** Causal relationship between SLE and AMD visualized through multiple analysis techniques including scatter plot, forest plot, leave-one-out plot, and funnel plot in the replication sample.

**
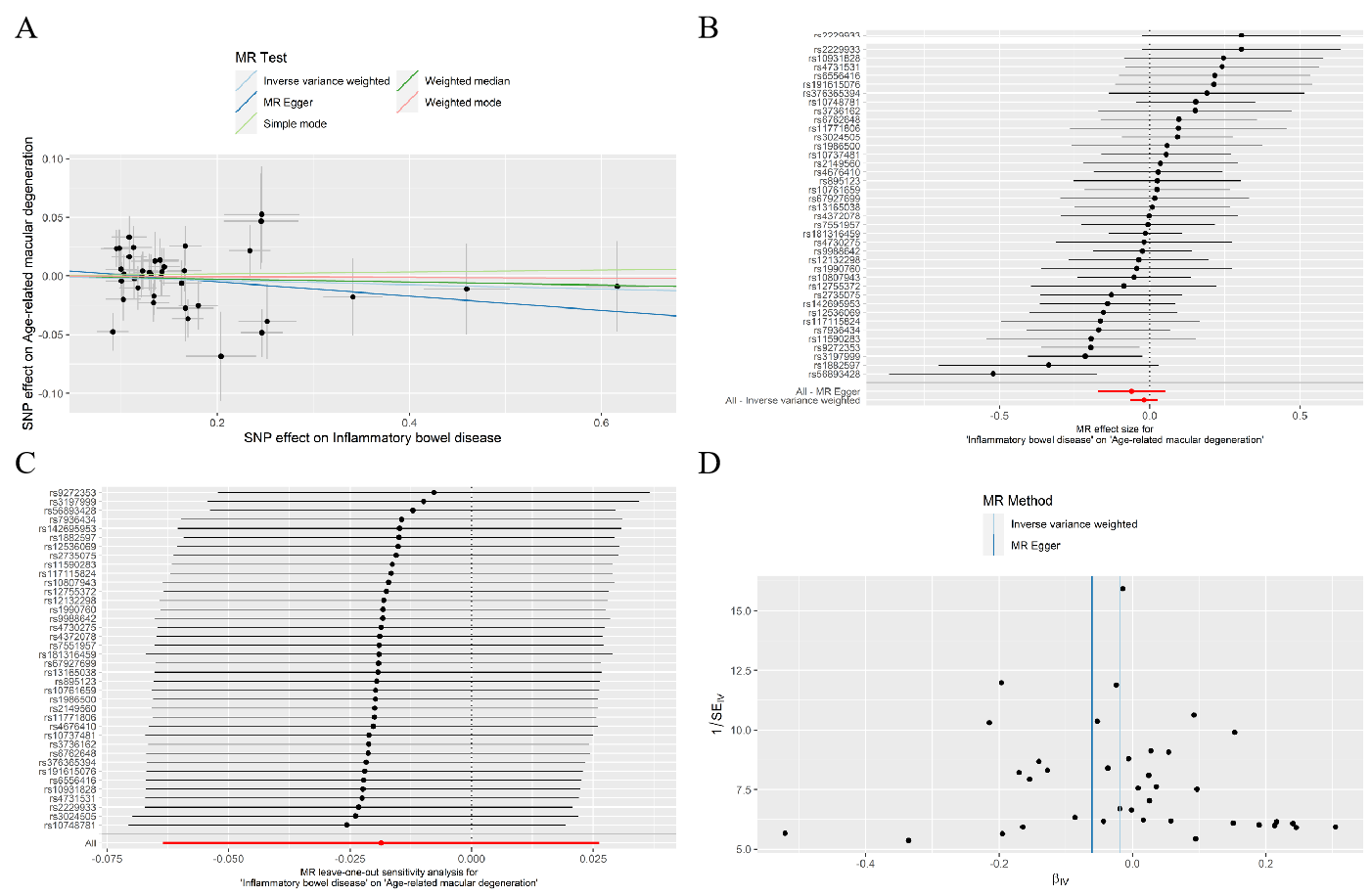
**

**Fig S8.** Causal relationship between SLE and AMD visualized through multiple analysis techniques including scatter plot, forest plot, leave-one-out plot, and funnel plot in the replication sample.

**
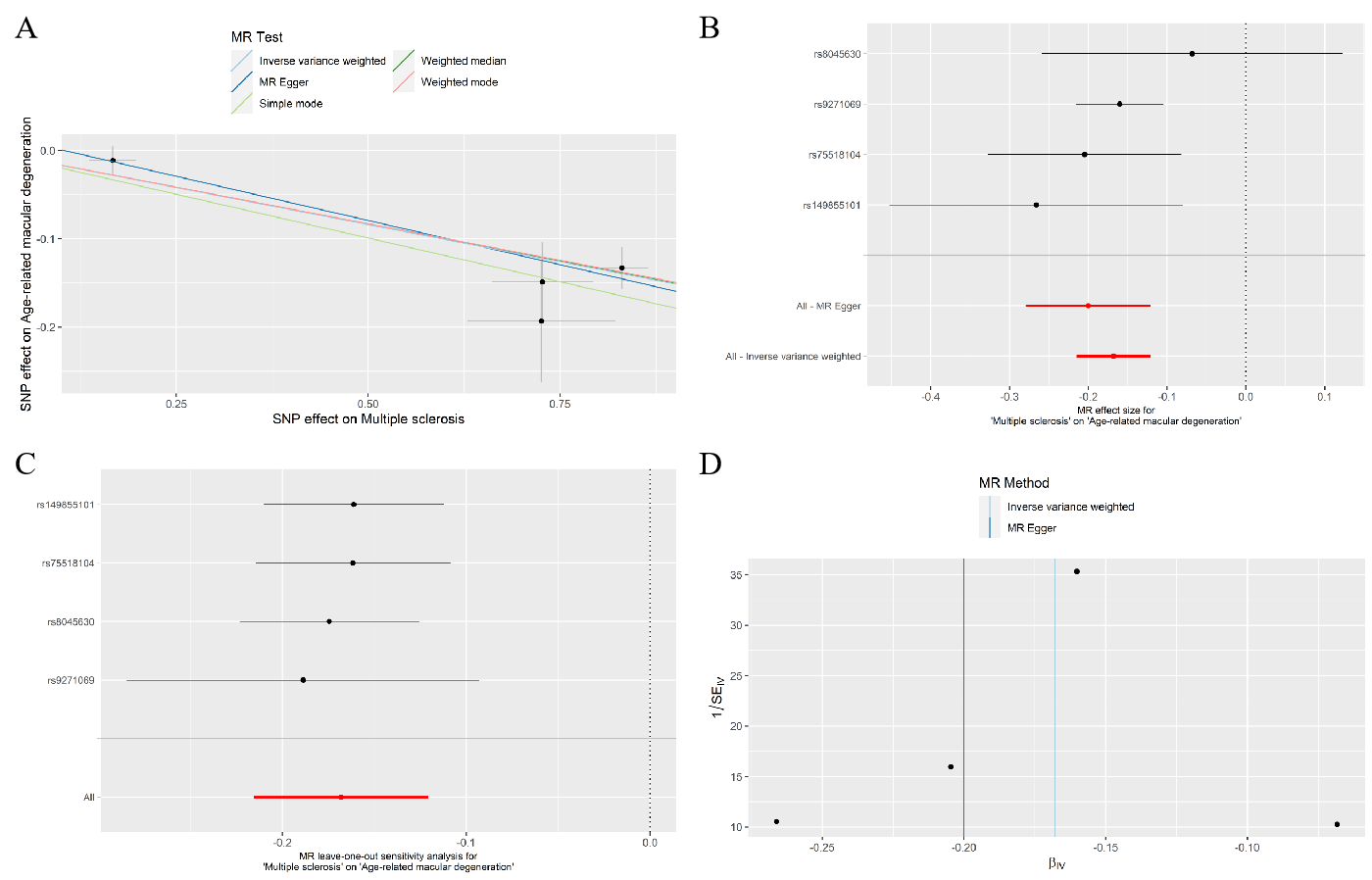
**

**Fig S9.** Causal relationship between SLE and AMD visualized through multiple analysis techniques including scatter plot, forest plot, leave-one-out plot, and funnel plot in the replication sample.

**
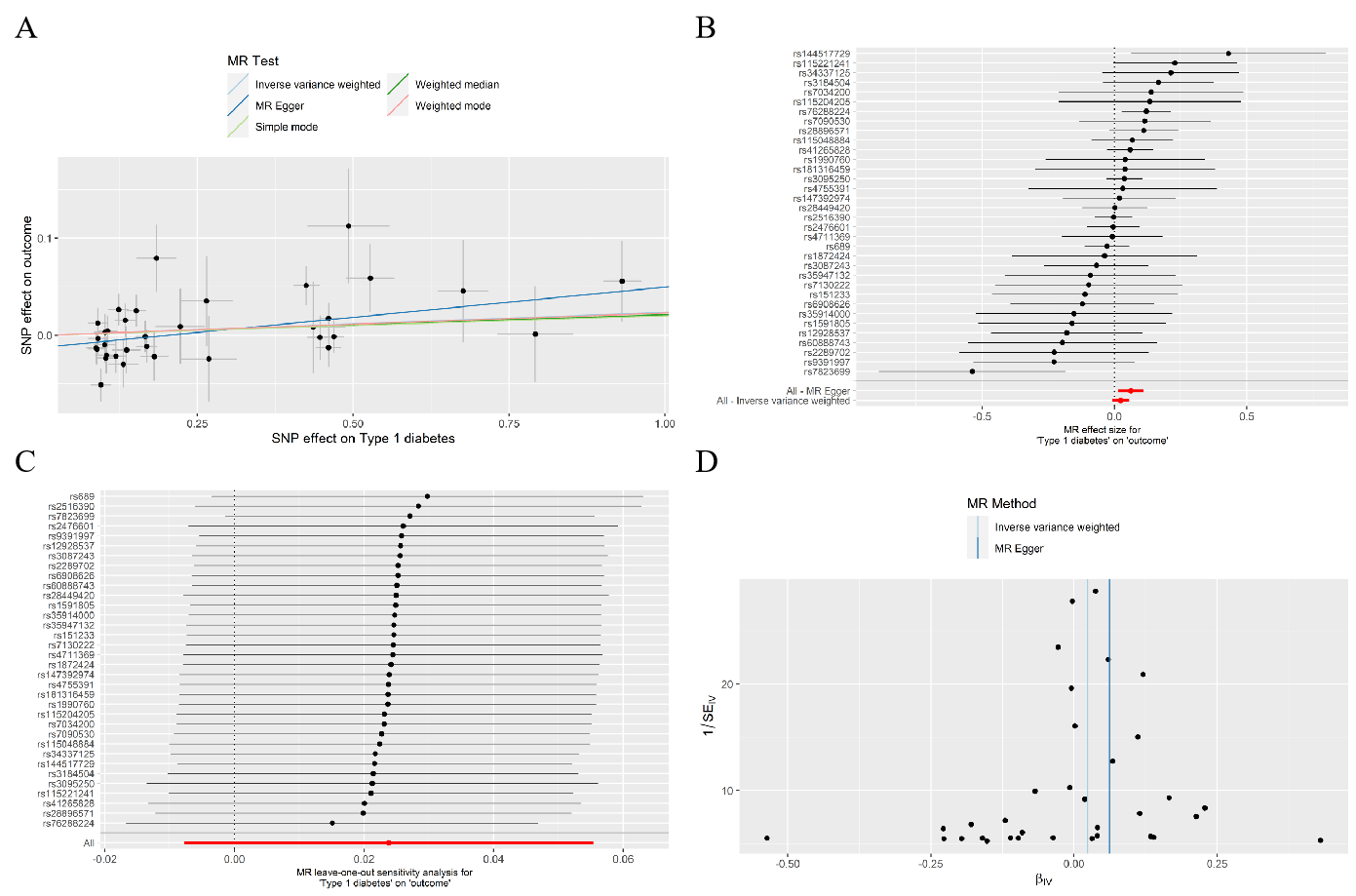
**

**Fig S10.** Causal relationship between SLE and AMD visualized through multiple analysis techniques including scatter plot, forest plot, leave-one-out plot, and funnel plot in the replication sample.
